# Circulating Cell Biomarkers in Pulmonary Arterial Hypertension: Relationship with Clinical Heterogeneity and Therapeutic Response

**DOI:** 10.3390/cells10071688

**Published:** 2021-07-04

**Authors:** Olga Tura-Ceide, Isabel Blanco, Jéssica Garcia-Lucio, Roberto del Pozo, Agustín Roberto García, Elisabet Ferrer, Isabel Crespo, Diego A. Rodríguez-Chiaradia, Carmen Pilar Simeon-Aznar, Manuel López-Meseguer, Clara Martín-Ontiyuelo, Víctor I. Peinado, Joan Albert Barberà

**Affiliations:** 1 Department of Pulmonary Medicine, Hospital Clínic-Institut d’Investigacions Biomèdiques August Pi i Sunyer (IDIBAPS), University of Barcelona, 08036 Barcelona, Spain; iblanco2@clinic.cat (I.B.); jss5@hotmail.com (J.G.-L.); rdelpozo81@gmail.com (R.d.P.); garcia83@clinic.cat (A.R.G.); elisabet.ferrer7@gmail.com (E.F.); clmartin@clinic.cat (C.M.-O.); vpeinado@clinic.cat (V.I.P.); 2 Biomedical Research Networking Centre on Respiratory Diseases (CIBERES), 28029 Madrid, Spain; darodriguez@pardesalutmar.cat; 3 Girona Biomedical Research Institute (IDIBGI), 17190 Girona, Spain; 4 Flow Cytometry and Cell Sorting Facility, Institut d’Investigacions Biomèdiques August Pi i Sunyer (IDIBAPS), 08036 Barcelona, Spain; isabel.crespo@idibaps.org; 5 Pulmonology Department, Hospital del Mar, Institut Hospital del Mar d’Investigacions Mèdiques (IMIM), Universitat Pompeu Fabra (UPF), 08003 Barcelona, Spain; 6 Unitat de Malaties Autoimmunes Sistèmiques. Servei de Medicina Interna Hospital Universitari Vall d’Hebron, Departament de Medicina, Universitat Autònoma de Barcelona, 08035 Barcelona, Spain; cpsimeon@vhebron.net; 7 Servei de Pneumologia, Hospital Universitari Vall d’Hebron, Departament de Medicina, Universitat Autònoma de Barcelona, 08035 Barcelona, Spain; manuelopez@vhebron.net

**Keywords:** pulmonary arterial hypertension, endothelial dysfunction, biomarkers, progenitor cells, endothelial extracellular vesicles, PAH-specific treatment

## Abstract

Background: Endothelial dysfunction is central to PAH. In this study, we simultaneously analysed circulating levels of endothelial microvesicles (EMVs) and progenitor cells (PCs) in PAH and in controls, as biomarkers of pulmonary endothelial integrity and evaluated differences among PAH subtypes and as a response to treatment. Methods: Forty-seven controls and 144 patients with PAH (52 idiopathic, 9 heritable, 31 associated with systemic sclerosis, 15 associated with other connective tissue diseases, 20 associated with HIV and 17 associated with portal hypertension) were evaluated. Forty-four patients with scleroderma and 22 with HIV infection, but without PAH, were also studied. Circulating levels of EMVs, total (CD31^+^CD42b^−^) and activated (CD31^+^CD42b^−^CD62E^+^), as well as circulating PCs (CD34^+^CD133^+^CD45^low^) were measured by flow cytometry and the EMVs/PCs ratio was computed. In treatment-naïve patients, measurements were repeated after 3 months of PAH therapy. Results: Patients with PAH showed higher numbers of EMVs and a lower percentage of PCs, compared with healthy controls. The EMV/PC ratio was increased in PAH patients, and in patients with SSc or HIV without PAH. After starting PAH therapy, individual changes in EMVs and PCs were variable, without significant differences being observed as a group. **Conclusion:** PAH patients present disturbed vascular homeostasis, reflected in changes in circulating EMV and PC levels, which are not restored with PAH targeted therapy. Combined measurement of circulating EMVs and PCs could be foreseen as a potential biomarker of endothelial dysfunction in PAH.

## 1. Introduction

Pulmonary arterial hypertension (PAH) is a rare and fatal disease characterized by a sustained increase in pulmonary vascular resistance that may lead to right ventricular failure and death [1,2]. The increase in pulmonary vascular resistance is associated with vessel wall remodelling in pre-capillary arteries and arterioles caused by the proliferation of resident and inflammatory cells [3]. These alterations are produced through different pathways, which result in endothelial injury and dysfunction [3]. Endothelial dysfunction, characterized by a downregulation of vasodilator and antiproliferative mediators and an overexpression of vasoconstrictor and proliferative agents, is a key event in the pathogenesis of PAH. In fact, current treatments act on these endothelial signalling pathways and could eventually go far beyond the vasodilator effect, influencing the balance between proliferation and apoptosis of vascular cells [4].

Treatment of PAH with targeted therapy has provided clear benefit in terms of survival, symptom relief, exercise tolerance and quality of life [5]. However, the eventual effects on vascular remodelling have been difficult to substantiate, and the pathological evaluation of the lungs of patients undergoing long-term treatment with targeted therapy has revealed the persistence of substantial vascular derangement [6]. The availability of biological cell markers that would inform the pathological processes that take place in pulmonary vessels, specifically the degree of remodelling and endothelial dysfunction, would allow for improved monitoring of disease progression and eventually treatment individualization.

The clinical characterization of PAH severity and the assessment of the response to treatment relies on the multiparameter assessment of one year mortality risk [7]. This multiparameter assessment involves clinical data, exercise tolerance, pulmonary haemodynamics, imaging studies and plasma brain natriuretic peptide (BNP) levels [8]. However, these measurements provide little information on the underlying biopathological process that gives rise to PAH, which is the alteration of the pulmonary endothelium.

Cellular biomarkers can reflect the status of the endothelial cells and provide information about its function [9]. These biomarkers include circulating microvesicles and progenitor cells. Endothelial microvesicles (EMVs) are small membrane vesicles with a diameter < 0.1 μm, released by endothelial cells in response to activation, damage and/or apoptosis [10]. Circulating EMVs can be identified and quantified in peripheral blood by flow cytometry [11]. In healthy individuals they are present at low levels, reflecting the normal turnover of endothelial cells, but they have been shown to increase cardiovascular disorders [12] and are associated with the clinical course of coronary disease [13]. In PAH, compared with control subjects, the levels of circulating EMVs increase and correlate with the severity of the disease [14].

Progenitor cells (PCs) are small immature cell precursors detectable in the blood. They are produced in the bone marrow and mobilized into circulation in response to different stimuli, to replace damaged endothelial cells [8,15,16]. At present, it is not clear if the number of circulating PCs differs between the different PAH subtypes, or whether it is modified by specific PAH therapy [15].

Previous studies in PAH have provided contradictory results. Whereas an increase of circulating PCs in patients with PAH has been reported in some studies [17], others have shown the opposite [18,19,20]. We hypothesize that in PAH, pulmonary vascular impairment would be associated with changes in the level of circulating cell markers of vascular competence, namely EMVs and PCs, and that their quantification would differ among different PAH subtypes and as a response to targeted treatment. The combined measurement of cellular markers of endothelial lesion (EMVs) and those reflecting the body’s regeneration capacity (PCs) emerges as a potential new approach to assess vascular competence [15] in PAH. Accordingly, the present study aimed to simultaneously analyse the number of circulating EMVs and PCs in patients with PAH and in control subjects, and to evaluate differences among PAH subtypes and as a response to treatment.

## 2. Materials and Methods

### 2.1. Study Population

This is an observational, case-control study comprising both cross-sectional and longitudinal studies. For the cross-sectional study, a total of 144 PAH patients with different PAH subtypes, including idiopathic (iPAH, n = 52), heritable (hPAH, n = 9), associated with systemic sclerosis (PAH-SSc, n = 31), associated with other connective tissue diseases (PAH-CTD, n = 15), associated with HIV (PAH-HIV, n = 20) and associated with portal hypertension (PAH-PoH, n = 17), were evaluated and compared with a group of 47 healthy control subjects (CL). In addition, we also analysed patients with underlying diseases at risk of developing PAH, namely scleroderma (SSc) (n = 44) and HIV infection (n = 22). In these patients, the presence of pulmonary hypertension was ruled out by normal Doppler echocardiography. The diagnosis of PAH was confirmed by right heart catheterization and other causes of pulmonary hypertension were excluded. Subjects with hemodynamic instability, severe comorbidities or lack of informed consent were excluded. The characteristics of patients with PAH, SSc and HIV and healthy control subjects are shown in Table 1. The absence of known lung diseases was confirmed by clinical evaluation and lung function testing. Heart diseases were also ruled out by clinical history and electrocardiogram.

In the cross-sectional study, 53 patients were treatment naïve and 91 prevalent patients received PAH therapy 29 a phosphodiesterase-5 inhibitor (PDE-5i), 23 an endothelin receptor antagonist (ERA), 23 combined treatments with ERA and PDE-5i, 5 combined treatments with PDE-5i and prostanoid, 6 triple combination therapy with ERA, PDE-5i and prostanoid and 5 calcium channel blockers (CCB). After diagnosis, treatment of incident patients was established by the attending physician according to international guidelines [7] and was not influenced by participation in the study. The longitudinal study included 53 PAH treatment-naïve patients. 22 patients started treatment with a PDE-5i, 12 with an ERA, 12 combined treatments with ERA and PDE-5i, and one patient started with triple combination therapy including ERA, PDE-5i and prostanoid. Six patients classified as PAH with acute vasoreactivity were treated with CCB.

### 2.2. Measurements

Subjects underwent standard evaluation by means of medical history, clinical examination, electrocardiogram and pulmonary function tests. Pre-capillary PH was considered when mean pulmonary arterial pressure ≥20 mmHg, pulmonary artery wedge pressure of ≤15 mmHg and pulmonary vascular resistance ≥250 dyn·s·cm^5^, measured at rest by right heart catheterization [1]. Other groups with PH rather than PAH were ruled out according to the current diagnostic algorithm [7]. Severity of PAH was assessed by doppler echocardiography, pulmonary haemodynamics, New York Heart Association-World Health Organization functional class (NYHA-WHO FC), 6-min walking distance (6-MWD) and blood brain natriuretic peptide (BNP) level measured at baseline and at follow-up.

Venous blood samples were obtained from fasting subjects by peripheral venepuncture and placed into two 4.5 mL sodium citrate tubes (Becton Dickinson, UK) to measure circulating EVs, and into two 4 mL tubes with EDTA (Becton Dickinson, UK) to measure circulating PCs. Samples were obtained in a different day or if in the same day before right heart catheterization.

### 2.3. Assessment of Circulating Endothelial Microvesicles (EMVs)

Circulating EMVs were assessed by flow cytometry by the expression of the platelet endothelium adhesion molecule-1 (PECAM-1, CD31) marker in the absence of the platelet-specific glycoprotein marker CD42b, as previously described by our group [15]. To further evaluate whether EMVs could derive from activated endothelial cells, EMVs were also assessed by CD62E (E-selectin) staining, a cell adhesion molecule expressed only on endothelial cells activated by cytokines. Peripheral blood was collected and within 1 h was centrifuged for 10 min (800× *g*, 4 °C) to prepare platelet rich plasma. Within 5 min, the supernatant was subsequently centrifuged for 10 min (300× *g*, 23 °C) to discard cells, 10 min (2000 × *g*, 23 °C) to discard dead cells and finally 30 min (10.000× *g*, 23 °C) to discard cell debris and obtain platelet-poor plasma (PPP). EMV phenotype analysis was performed based on size and fluorescence. Events less than 1 μm were identified in forward (size) and side (density) light scatter plots using size calibration microspheres (FluoSpheres 1 carboxylate-modified microspheres 1.0 μm, yellow-green fluorescent (505/515), Invitrogen, Oregon, EEUU). EMVs levels were assessed by comparison with calibrator beads (Perfect Count Microspheres, Cytognos, Salamanca, Spain) with a known concentration, using 2.000 events beads-PE as a stop time. Then 100.000 MPs/μL for fluorescent minus one (FMO) tubes and 500.000 MPs/μL for each phenotype were stained for 45 min at room temperature using pre-conjugated anti-human monoclonal antibodies and isotype controls: anti-CD31-FITC (BD PharmingenTM, San Jose, CA, USA), anti-CD42b-PE (BD PharmingenTM, San Jose, CA, USA), anti-CD62E-APC (BD PharmingenTM, San Jose, CA, USA), anti-IgG1 k-PE isotype control and anti-IgG1k-APC isotype control, both from (BD PharmingenTM, San Jose, CA, USA) for the activated phenotype. The fluorescence minus one technique was employed to provide negative controls [21]. Samples were analysed by two- or three-color fluorescence histograms as CD31^+^CD42b^−^ or CD31^+^CD42b^−^CD62E^+^ microparticles. Single antibody conjugates and compensation fluorochrome beads were used for compensation assessment. Samples were acquired at band pass filters: 530 nm (FITC), 585 nm (PE/PI) and 661 nm (APC) with FL4 option. EMVs were quantified by flow cytometry using LRSFortessa (BD Bioscience, San Jose, CA, USA) and 100.000 MPs/events were acquired. The gating strategy is shown in Supplementary Figure S1. The data were analysed using FACSDIVA (Tree Star, OR, USA).

### 2.4. Evaluation of Circulating Progenitor Cells (PCs)

The number of circulating progenitor cells was evaluated by flow cytometry using antibodies against CD45 (pan-leukocyte marker), CD133 (sub-population of hematopoietic stem cells) and CD34 (mature and progenitor endothelial cells) as previously described [15]. In brief, circulating PCs were isolated from fresh peripheral blood by Ficoll density gradient centrifugation, washed once with phosphate buffered saline (PBS) supplemented with 2% of foetal calf serum (FCS) and resuspended at 2 × 10^6^ cells for FMO tubes and at 4 × 10^6^ cells for sample tubes. Circulating PCs were stained and analysed by flow cytometry for phenotypic expression of surface markers using pre-conjugated anti-human monoclonal antibodies and isotype controls anti-CD45-FITC (BD Pharmingen TM, San Jose, CA, USA), anti-CD34-PECy7 (eBiosciences, San Diego, CA, USA), anti-CD133-PE (MACS Miltenyi Biotec, Bergisch Gladbach, Germany), anti-IgG1k-PECy7 isotype control (eBiosciences, San Diego, CA, USA), anti-IgG1k-FITC isotype control (BD PharmingenTM, San Jose, CA, USA) and anti-IgG1k-PE isotype control (BD PharmingenTM, San Jose, CA, USA). The fluorescence minus one technique was employed to provide negative controls [21]. After 45 min of incubation, cells were washed, resuspended in 500 μL of PBS +2% FCS and subjected to flow cytometry analysis, LRSFortessa (BD Bioscience, San Jose, CA, USA). The data were analysed using FACSDIVA (Tree Star, OR, USA) following ISHAGE (International Society of Hematotherapy and Graft Engineering) as previously published [22]. Gating strategy shown in Supplementary Figure S1.

### 2.5. Statistical Analysis

Data are expressed as mean ±SD or mean and 95% confidence interval. Pairwise comparisons between patients and controls were performed using Student’s *t*-test for normally distributed variables, or Mann Whitney Rank Sum test for non-normally distributed variables. Comparisons involving more than two groups were performed with Kruskal-Wallis One Way Analysis of Variance and when significant, post-hoc Dunn’s multiple comparisons test were performed. Correlations between variables were analysed with Pearson’s or Spearman´s coefficient depending on data distribution. A *p* value < 0.05 was considered significant in all cases.

## 3. Results

### 3.1. Population Characteristics

Anthropometric, clinical and functional characteristics of patients and controls are shown in Table 1. Compared with healthy controls, patients with PAH were slightly older and showed reduced spirometric values, although within normal range in the majority of cases, and reduced CO diffusing capacity (DLco). Patients with SSc were mostly females and showed reduced FEV_1_/FVC ratio, DLco and higher BNP. HIV patients were mostly males and presented lower FEV_1_/FVC ratio than control subjects (Table 1). Table 2 shows the characteristics of patients with different PAH subtypes. Patients with PAH-SSc showed the lowest DLco and mPAP values, compared with the other PAH subtypes (Table 2).

### 3.2. Circulating Endothelial Microvesicles (EMVs)

**Figure 1:**
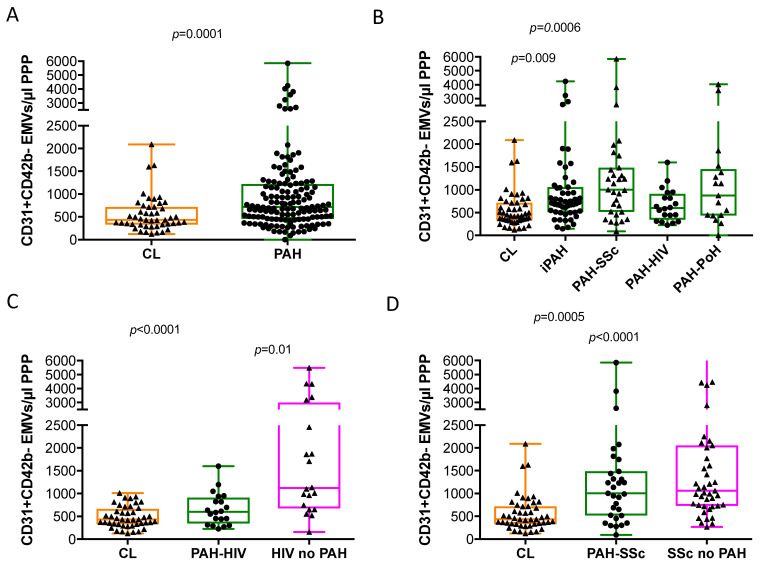
Number of circulating endothelial microvesicles (EMVs) in pulmonary arterial hypertension, scleroderma and HIV infection and healthy controls. Number of (**A**) CD31^+^CD42b^−^ endothelial microvesicles (EMVs), expressed per μL of poor platelet plasma, in pulmonary arterial hypertension (PAH) versus healthy controls; (**B**) number of CD31^+^CD42b^−^ EMVs in all the different PAH subtypes versus healthy controls; (**C**) number of CD31^+^CD42b^−^ EMVs in patients with, HIV infection or without associated PAH; (**D**) number of CD31^+^CD42b^−^ EMVs in patients with scleroderma, with or without associated PAH. The whiskers extend from the minimum to maximum points. Mann Whitney Rank Sum test and Kruskal-Wallis One Way Analysis of Variance and when significant, post-hoc Dunn’s multiple comparisons test.

Patients with PAH showed significantly higher numbers of EMVs compared with healthy controls (Figure 1A and Table 3). When analysed by PAH subtypes, patients with iPAH and PAH-SSc showed a significantly higher number of circulating EMVs than healthy controls, without significant differences between them (Figure 1B). The number of EMVs derived from activated endothelial cells (CD31^+^CD42b^−^CD62E^+^) in PAH did not differ from healthy controls; only the group of patients with PAH-SSc showed a higher number of activated EMVs than controls (Table 3). Interestingly, whereas patients with PAH-HIV had similar numbers of EMVs to healthy controls, HIV patients without PAH had a significantly higher number of EMVs than both healthy controls and PAH-HIV patients (Figure 1C). The increased number of circulating EMVs in patients with HIV but without PAH was not due to activated EMVs, because activated EMVs were less abundant than in healthy controls (Table 3). Furthermore, patients with SSc, with or without PAH, showed higher numbers of EMVs than the healthy controls (Figure 1D and Table 3).

### 3.3. Circulating Progenitor Cells (PCs)

**Figure 2:**
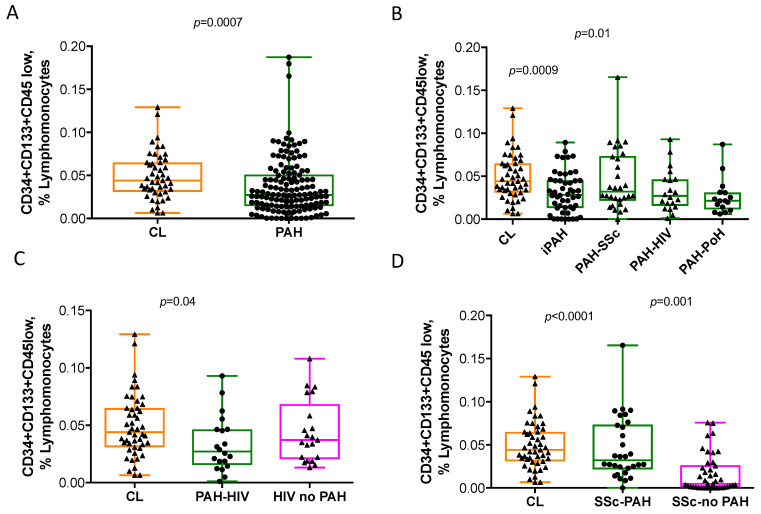
Percentage of circulating progenitor cells (PCs) in pulmonary arterial hypertension, scleroderma and HIV infection and healthy controls. Percentage of (**A**) CD34^+^CD133^+^CD45^low^ labelled cells, expressed as percent of lymphomonocytes, in pulmonary arterial hypertension (PAH) versus healthy controls; (**B**) percentage of CD34^+^CD133^+^CD45^low^ labelled cells in all the different PAH subtypes versus healthy controls; (**C**) Percentage of CD34^+^CD133^+^CD45^low^ labelled cells in patients with HIV infection, with or without associated PAH; (**D**) percentage of CD34^+^CD133^+^CD45^low^ labelled cells in patients with scleroderma, with or without associated PAH. The whiskers extend from the minimum to maximum points. Mann Whitney Rank Sum test and Kruskal-Wallis One Way Analysis of Variance and when significant, post-hoc Dunn’s multiple comparisons test.

Patients with PAH showed significantly lower percentages of PCs, defined as CD34^+^CD133^+^CD45^low^, compared with healthy controls (Figure 2A and Table 3). When assessing differences among PAH subtypes, patients with iPAH and PAH-PoH showed significantly lower percentages of circulating PCs than healthy controls (Figure 2B). Patients with PAH-HIV showed a reduced percentage of PCs compared with healthy subjects, whereas patients with HIV but without PAH did not differ from controls (Figure 2C). Additionally, whilst patients with PAH-SSc presented similar levels of PCs than controls, those with SSc but without PAH had significantly lower levels of PCs than healthy subjects and patients with PAH-SSc (Figure 2D).

### 3.4. EMVs/PCs Ratio

**Figure 3:**
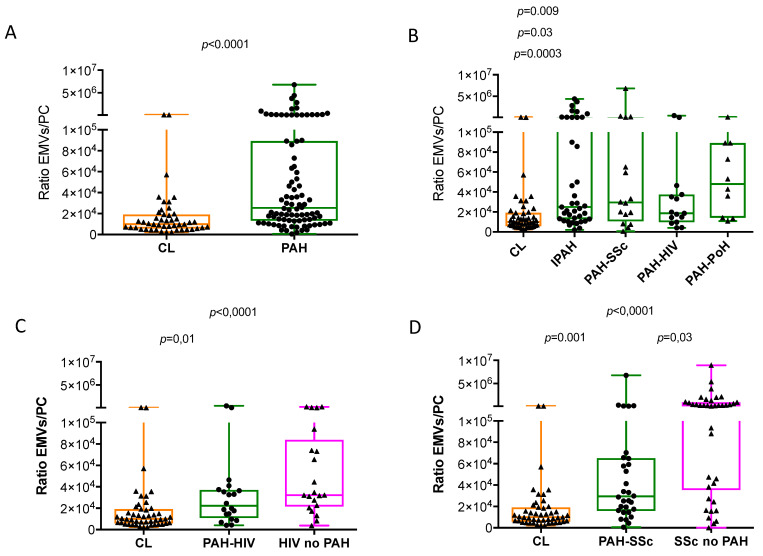
Ratio of EMVs/PCs in pulmonary arterial hypertension, scleroderma and HIV infection and healthy controls.Ratio between CD31^+^CD42b^−^ endothelial microvesicles, expressed per μL of poor platelet plasma and percentage of CD34^+^CD133^+^CD45^low^ labelled cells (EMVs/PCs ratio) in (**A**) patients with pulmonary arterial hypertension (PAH) versus healthy controls; (**B**) all the different PAH subtypes versus healthy controls; (**C**) patients with HIV infection, with or without associated PAH; (**D**) patients with scleroderma, with or without associated PAH. Mann Whitney Rank Sum test and Kruskal-Wallis One Way Analysis of Variance and when significant post-hoc Dunn’s multiple comparisons test.

We assessed the ratio of EMVs to PCs, which reflects the balance between endothelial cell damage and repair capacity as a measure of vascular competence. The EMVs/PCs ratio was significantly increased in patients with PAH and in most PAH subtypes, compared with healthy individuals (Figure 3A,B). Patients with HIV, with or without PAH, had significantly higher ratios of EMV/PC than controls (Figure 3C). Likewise, SSc patients, with or without PAH, showed a significantly higher ratio than controls; the group of SSc patients without PAH showed the highest ratio (Figure 3D).

No significant association between the number of circulating EMVs with PC was observed (Figure S2).

Additionally, age-matched subanalyses were performed in PAH subtypes and controls. Differences in the levels of EMVs, PCs and the EMVs/PCs ratio remained unchanged with respect to the whole group (Supplementary Figure S3). Since there were differences in gender distribution in HIV and SSc patients with and without PAH (Supplementary Table S1), we performed an exploratory gender-matched analysis. Differences between groups in EMVs and EMVs/PCs ratio were the same (data not shown).

### 3.5. Changes Induced by PAH Treatment

Despite the improvement of clinical features, there were no significant differences in the numbers of circulating EMVs, PCs and the EMVs/PCs after 3 months, regardless of the type of therapy used (Supplementary Table S2). Basal risk parameters analysed (6MWD, NYHA-FC, BNP) and hemodynamic parameters were not correlated with pre-treatment level of neither EMVs nor PCs (Supplementary Table S3). Additionally, the differences found in circulating PCs and EMVs levels in HIV and SSc patients with and without PAH, were not related to PAH-targeted treatment (Supplementary Figure S4).

**Figure 4:**
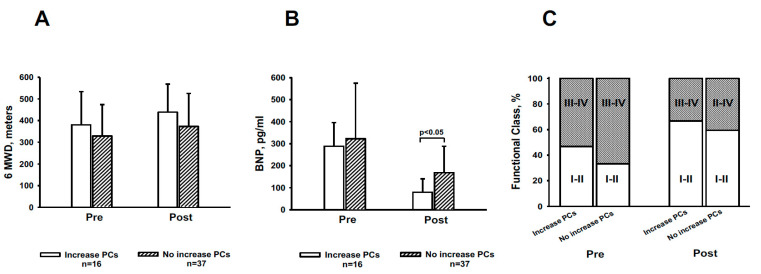
Clinical effects of treatment according to the change induced in circulating progenitor cells. Values of six-minute walking distance (6MWD) (**A**), brain natriuretic peptide (BNP) (**B**) and functional class (**C**) in 53 patients with pulmonary arterial hypertension (PAH), before (Pre) and 3 months after (Post) initiating targeted PAH therapy. Patients were stratified according to the change in CD34^+^CD133^+^CD45^low^ labelled cells (PCs) after treatment. Open bars show values of patients who showed an increase in circulating PCs, n = 16 dashed bars show values of patients showing a decrease or no change in circulating PCs, n = 37.

However, the individual responses were variable. While the number of circulating EMVs or PCs has increased in some patients, both remained stable or decreased in others. Interestingly, increase in the levels of PC following initiation of PAH treatment was associated with improved exercise tolerance and a significant decrease in BNP levels (Figure 4), thereby suggesting an improvement in cardiac function. In addition, few patients underwent right heart catheterization after treatment initiation (n = 12). Cardiac index was significantly improved only in the subgroup of patients who showed an increase in circulating PCs after starting targeted-PAH therapy (Supplementary Table S4).

## 4. Discussion

The main findings of the present study are that patients with PAH show increased levels of circulating EMVs and a decreased number of circulating PCs, with an imbalance in their ratio. These changes were especially evident in idiopathic PAH, PAH associated with scleroderma and PAH-associated with portal hypertension. Patients who increased the number of circulating PCs after starting targeted PAH therapy showed a more favourable clinical risk profile than those in whom the number of circulating PCs did not increase or decreased.

Patients with PAH in our series showed a circulating biomarker profile compatible with endothelial dysfunction, with increased numbers of circulating EMVs, a marker of endothelial damage [23,24,25] and a reduced number of circulating PCs, suggesting reduced capacity for endothelial cell repair [9,15]. The ratio between EMVs and PCs, which has been considered a marker of vascular competence [15,23], was significantly increased in patients with PAH.

It has been suggested that EMVs might play a role in the pathogenesis of pulmonary hypertension [24]. Amabile et al. reported an increased number of circulating EMVs in a mixed group of patients with different pulmonary hypertension aetiologies, including PAH and chronic pulmonary disease–related pulmonary hypertension, which correlated to disease severity [14,25]. Additionally, in PAH, EMVs have been considered key modulators in inflammatory and coagulation PAH-associated processes [26,27,28].

Our results confirm the increase of circulating EMVs in PAH, Additionally, we aimed to analyse whether the number of EMVs differ among different PAH subtypes. We also included patients with diseases at risk of developing PAH, namely scleroderma and HIV infection, to evaluate to what extent differences in circulating EMVs could be related to the underlying diseases. Our results show that the levels of EMVs vary between the different PAH subtypes, being significantly increased, compared to healthy controls, in iPAH and PAH-SSc. Matched PAH and controls subanalyses suggest that the observed results found are independent of gender or age.

To date, the cellular mechanisms behind this EMV increase in PAH are not fully understood. In our series, PAH patients presented a significant rise in CD31^+^CD42b^−^ but not in CD31^+^CD42b^−^CD62E^+^ EMVs, the latter reflecting EMVs derived from activated endothelial cells. Accordingly, our results suggest that circulating EMV augmentation in PAH seems to result from an increment of endothelial damage/apoptosis rather than an increase of endothelial cell activation. PAH is a vascular disease involving pulmonary vasoconstriction, vessel remodelling and obliteration, and the development of plexiform lesions in which endothelial cells play a detrimental role [29]. It has been postulated that in pulmonary hypertension, EMVs are indicative of cell injury facilitating cell proliferation, inflammation, altered angiogenesis and apoptosis [24]. Additionally, Blair et al. have shown that circulating microvesicles induce an increase in ICAM-1, which facilitates the recruitment of inflammatory infiltrates to the pulmonary arteries [30]. Whether the release of EMVs triggers PAH pathogenesis and the advance of the disease, or it is a consequence of the disease inherent vascular damage is unknown. Our results seem to suggest that they are more likely a marker of endothelial injury.

Scleroderma is a disease characterized by an abnormal endothelial cell lining and inflammation, commonly associated with PAH development [31]. The number of microparticles in SSc patients has been studied, albeit with contradictory results. Whilst some studies have shown a strong increase in EMV in patients with SSc compared to controls [31,32,33], others have found opposite results [34,35]. Recently Lammi et al. [31] showed a higher number of EMVs in SSc-PAH compared to patients with SSc without PAH and controls. Interestingly, EMVs isolated from patients with SSc had inflammatory inducing properties for healthy endothelial cells [31]. In our series, patients with SSc, regardless of whether they had PAH or not, showed an increased number of EMVs, measured by the CD31 endothelial marker in the absence of the platelet-specific glycoprotein marker CD42b.

Overall, these results elucidate the presence of endothelial injury in SSc irrespective of the severity of pulmonary vascular involvement, and that EMVs can be recorded and followed by flow cytometry quantification. This posits EMVs as a potentially novel biomarker to enhance patient early diagnosis and disease treatment monitoring.

Even though not a target of the virus, endothelial proatherogenic cell dysfunction is a ubiquitous event present in patients with HIV-1 infection [36]. Factors causing such endothelial dysfunction are not yet well understood. There are scarce studies on the role of EMVs in HIV infection. Recent studies show that EMVs, identified as CD62E^+^, are significantly increased in patients with HIV infection treated with antiretroviral therapy when compared to controls [36]. Additionally, Hijmans et al. showed that HIV-1 proteins are able to induce EMV release from endothelial cells and to promote endothelial senescence, inflammation and oxidative stress [37].

Our study shows that while EMV levels were increased in all the HIV patients, those with HIV-associated PAH, had similar EMV levels to the healthy subjects. Accordingly, the HIV infection disease itself might induce vascular damage. In fact, certain endothelial beds have been shown to be particularly vulnerable to HIV infection, enhancing endothelial cell adhesion, proliferation, apoptosis and tissue remodelling [38]. These pathogenic induced mechanisms might translate to an abnormal release of EMV into the body’s patient circulation. In addition, the number of activated EMVs (CD31^+^CD42b^−^CD62^+^) was downregulated in HIV, with or without PAH, when compared with healthy controls. This observation suggests that the rise of EMVs in HIV does not appear to be due to endothelial cell activation, but to increased vascular damage or apoptosis.

Bone marrow mobilizes and releases progenitor cells (PCs) into the blood, contributing to the body’s endothelial injury repair [15,16]. In PAH, studies carried out to date have yielded conflicting results. While an increase in circulating PCs has been observed in some [17], others have found a reduced number [18,19,20]. These discrepancies could be explained by differences in the markers used, methodological divergences or the severity of the disease. Our group has previously shown in a small group of untreated PAH patients that the number of circulating PCs was decreased compared to healthy individuals [20]. The current study confirms our previous results and shows that the decrease in circulating PCs is consistent across the different PAH-subtypes and is irrespective of treatment. Whether PC deficit is inherent to patients and predisposes them to develop the disease or stems from an exhaustion of the PC bone marrow reservoir due to the patient’s continuous endothelial damage and vascular remodelling, is not yet resolved. Nevertheless, our results imply that regardless of PAH aetiology, impaired endothelial repair capacity is common among different PAH subtypes.

Patients with SSc who have suffered the disease for more than 5 years present a reduced number of circulating endothelial progenitor cells [39]. In our series, patients with SSc, with or without PAH, showed reduced levels of PCs compared to control subjects. Since SSc is associated with intrinsic angiogenic alterations [39], it is conceivable that this impairment could be due to progenitor cell insufficiency. Similarly, patients with PAH-HIV also presented a lower number of PCs compared to healthy subjects. However, patients with HIV but without PAH did not differ from healthy controls, thus suggesting that the appearance of PAH in patients with HIV further deprives endothelium repair capabilities.

Taken together, our results indicate that the assessment of the balance between the number of circulating EMVs and PCs can characterize a phenotype of disturbed vascular competence [15]. The imbalance between endothelial damage and repair capacity was present in all the PAH subtypes and can be a marker of impaired endothelial function in PAH. Of note, our study shows a lack of association between the number of circulating EMVs and the number of PC. This suggests that both cellular biomarkers measure independent vascular disturbances. Accordingly, the simultaneous measurement of both biomarkers in PAH provides more comprehensive information on endothelial cell status and function.

Current targeted PAH therapy aims to restore vascular homeostasis between vasoactive and vasoconstrictive endothelium-derived mediators. Conceivably, after treatment, EMV and PC levels might readjust and become similar to those observed in healthy subjects. In our series, the assessment of both EMV and PC levels after 3 months of initiating targeted PAH therapy did not change significantly considering all patients together. Targeted PAH therapy does not seem to have the capacity to entirely correct the vascular dysfunction of patients, which might be consistent with the fact that targeted PAH therapy is not curative. However, the individual response in circulating biomarkers was variable with respect to the change in the number of circulating EMVs or PCs. Accordingly, we analysed whether patients showing an improvement in the number of circulating biomarkers, namely a decrease in EMVs or increase in PCs after starting targeted PAH therapy, had more favourable clinical responses. Interestingly, patients who increased the number of circulating PCs had a more favourable clinical risk profile in terms of exercise tolerance, BNP levels and functional class, as compared with patients in whom circulating PCs decreased or remained unchanged. Even though the association was weak, this observation points out that patients with PAH showing increased levels of circulating PCs with targeted PAH therapy improved their endothelial cell status. To what extent the improvement in circulating PCs levels persists over time, correlates with the magnitude of clinical improvement, and is associated with a particular class of targeted PAH treatment remains to be elucidated.

The study has some limitations. First, as we sampled EMVs in peripheral venous blood we cannot be certain that the elevation of EMVs is from pulmonary origin. Further studies using specific markers to distinguish the origin of such EMVs should clarify this question. Second, healthy controls were slightly younger than the overall PAH population. However, when age-matched comparisons between PAH subtypes and healthy control subjects were performed, the results and main conclusions remained unchanged. Accordingly, age difference does not explain the observed results. Finally, there is no worldwide exclusive methodology to isolate and analyse circulating EMVs and PCs from peripheral blood, although we have used an updated and validated methodology.

In conclusion, this study shows that patients with PAH present a disturbed vascular homeostasis with an imbalance between endothelial damage (EMV) and repair capacity (PC), which is consistent among the different PAH subtypes. Accordingly, the combined measurement of circulating EMVs and PCs could be foreseen as a potential biomarker of endothelial dysfunction in PAH.

## Figures and Tables

**Table 1:** Clinical and hemodynamic characteristics between pulmonary arterial hypertension patients and controls.

Variables	All PAH n = 144	CL n = 47	SSc n = 44	HIV n = 22
Age, years	53.8 ± 15.2 *	48.0 ± 14.3	53.9 ± 10.1	43.3 ± 9.3
Male sex n (%)	41 (28.3%)	20 (42.5%)	5 (11.3%) ^†^	19 (86.3%) ^#^
BMI (Kg/m^2^)	26.4 ± 5.7	25.3 ± 4.0	25.7 ± 4.4	25.3 ± 4.0
FVC (% predicted)	95.6 ± 17.4 *	101.3 ± 12.6	100.4 ± 15.3	105.8 ± 13.9
FEV_1_ (% predicted)	81.4 ± 18.3 *	102.8 ± 13.2	101.3 ± 12.8	97.6 ± 16.2
FEV_1_/FVC (%)	74.4 ± 7.5 *	83.6 ± 6.0	79.0 ± 5.3 ^†^	74.5 ± 8.1 ^#^
TLC (% predicted)	89.7 ± 14.4 *	103.2 ± 10.6	103.1 ± 16.0	105 ± 13.3
DLco (% predicted)	52.7 ± 18.8 *	91.9 ± 14.6	73.4 ± 12.2 ^†^	87.8 ± 9.4
DLCO/VA	62.5 ± 18.8 *	101 ± 21.9	78.3 ± 12.5 ^†^	86.5 ± 14.4
PaO_2_ (mmHg)	67.5 ± 13.1	ND	ND	ND
mPAP (mmHg)	54.6 ± 17.4	ND	ND	ND
PAWP (mmHg)	10.2 ± 6.6	ND	ND	ND
RAP (mmHg)	8.2 ± 4.0	ND	ND	ND
CI (L/min/m^2^)	2.5 ± 0.7	ND	ND	ND
PVR (dyn·seg·cm^5^)	810.2 ± 465.1	ND	ND	ND
BNP (pg/mL)	324.1 ± 388.6 *	16.8 ± 4.3	47.9 ± 51.5 ^†^	18.5 ± 22.6
WHO FC n, (%)				
I	22 (15.1) *	47 (100)	43 (97.7)	18 (81.1) ^#^
II	57 (39.3)	0 (0)	0 (0)	2 (9.0)
III-IV	54 (37.2)	0 (0)	1 (2.2)	2 (9.0)

Data are shown as mean ± SD. Definition of abbreviations: PAH: pulmonary arterial hypertension; CL: healthy controls; BMI: body mass index; FVC: forced vital capacity; FEV_1_: forced expiratory volume in the first second; TLC: total lung capacity; DLco: diffusing capacity of the lung for carbon monoxide; PaO_2_: arterial partial oxygen pressure; mPAP: mean pulmonary arterial pressure; PAWP: pulmonary artery wedge pressure; RAP: right atrial pressure; CI: cardiac index; PVR: pulmonary vascular resistance; BNP: brain natriuretic peptide, WHO FC: functional class world health organization; and ND: not determined. * *p* < 0.05 PAH compared to controls, ^†^ *p* < 0.05 SSc compared to CL, ^#^ *p* < 0.05 HIV compared to CL.

**Table 2:** Clinical characteristics of patients with the different subtypes of pulmonary arterial hypertension.

Variables	iPAH n = 52	PAH-SSc n = 31	PAH-CTD n = 15	PAH-HIV n = 20	PAH-PoH n = 17
Age, years	52.8 ± 16.4	64.4 ± 11.2 *	50.1 ± 16.4 ^#^	46.2 ± 6.7 ^#^	56.5 ± 12.2
Male sex n (%)	20 (38.4%)	3 (9.6%)	2 (13.3%)	10 (50%)	7 (41.1%)
BMI (Kg/m^2^)	27,9 ± 6.0	26.3 ± 4.7	26.7 ± 5.3	23.0 ± 4.3 *^,$^	28.3 ± 5.2
FVC (% predicted)	86.6 ± 15.6	78.3 ± 18.9	81.8 ± 15.6	94.0 ± 16.2	88.0 ± 22.9
FEV_1_ (% predicted)	81.5 ± 16.3	77.3 ± 19.5	79.5 ± 17.1	83.5 ± 17.9	79.8 ± 20.5
FEV_1_/FVC (%)	72.7 ± 9.1	73.6 ± 6.4	76.1 ± 10.7	70.0 ± 9.9	70.4 ± 6.5
TLC (% predicted)	94.2 ± 10.3	84.2 ± 15.7	82.6 ± 13.0 ^†^	98.7 ± 12.6	94.9 ± 17.9
Dlco (% predicted)	56.7 ± 22.0	48.2 ± 16.8 *	50.0 ± 19.7	53.1± 9.9 ^#^	55.0 ± 15.0 ^#^
DLCO/VA	64.8 ± 23.6	63.9 ± 14.4	63.9 ± 21.9	58.6 ± 16.7	63.6 ± 13.3
PaO_2_ (mmHg)	74.4 ± 18.2	63.9 ± 14.4	71.9 ± 19.9	76.5 ± 14.2	71.6 ± 13.1
PAPm (mmHg)	53.8 ± 14.5	42.4 ± 13.5 *	44.6 ± 11.5	51.3 ± 13.7	50.2 ± 16.0
PAWP (mmHg)	9.2 ± 6.7	9.4 ± 3.1	7.1 ± 3.3	9.2 ± 4.3	9.7 ± 3.0
RAP (mmHg)	8.0 ± 5.0	9.3 ± 5.6	6.2 ± 3.0	7.4 ± 4.4	6.0 ± 2.7
CI, (L·m^2^·min^−1^)	2.2± 0.6	2.3± 0.5	2.5± 0.7	2.3± 0.7	2.7± 0.6 *
PVR (dyn·seg·cm^5^)	911.3 ± 373.4	682.3 ± 361.5	890.0 ± 503.3	894.5 ± 573.3	710.5 ± 360.3
BNP (pg/mL)	251.9 ± 337.9	327 ± 314.2	166.1 ± 255.9	199.5 ± 296.5	134.1 ± 173.7
WHO FC n, (%)					
I	13 (25.0)	1 (3.2) *	2 (13.3)	3 (15.0)	3 (17.6)
II	22 (42.3)	11 (35.4))	6 (40.0)	11 (55.0)	9 (52.9)
III-IV	17 (32.7)	19 (61.3)	7 (46.6)	6 (30.0)	5 (29.4)

Data are shown as mean±SD. Definition of abbreviations: PAH: pulmonary arterial hypertension, PAH-SSc: PAH-associated with systemic sclerosis; HIV-PAH: PAH-associated with HIV; CTD-PAH: PAH-associated with other connective tissue diseases; PoH-PAH: portopulmonary hypertension; BMI: body mass index; FVC: forced vital capacity; FEV_1_: forced expiratory volume in the first second; TLC: total lung capacity; DLco: diffusing capacity of the lung for carbon monoxide; PaO_2_: arterial partial oxygen pressure; mPAP: mean pulmonary arterial pressure; PAWP: pulmonary artery wedge pressure; CI: cardiac index; PVR: pulmonary vascular resistance; BNP: brain natriuretic peptide; FC WHO: functional class world health organization; and ND: not determined. * *p* < 0.05 PAH compared to iPAH, ^#^ *p* < 0.05 compared with PAH-SSc, ^†^ *p* < 0.05 compared with PAH-HIV, ^$^ *p* < 0.05 compared to PoH.

**Table 3:** Circulating endothelial microvesicles and progenitor cell counts.

Patients and Controls (mean, (95% CI)	EMVs/µL PPP CD31^+^CD42b^−^	Activated-EMVs CD31^+^CD42b^−^CD62E^+^	Lymphomonocytes, ×10^5^ Events	PCs CD34^+^CD133^+^ CD45^low^%, Lymphocytes	Ratio EMVs/PCs
Healthy controls n = 47	564 (447.7, 680.2)	18.5 (12.3, 24.6)	9.1 × 10^5^ (8.3 × 10^5^, 9.9 × 10^5^)	0.04 (0.04, 0.05)	1.8 × 10^4^ (1 × 10^4^, 2.5 × 10^4^)
All PAH n = 144	973.6 (830.5, 1117) *	23.8 (15.5, 32.1)	8.5 × 10^5^ (8.0 × 10^5^, 8.9 × 10^5^)	0.03 (0.03, 0.04) *	29.6 × 10^4^ (8.5 × 10^4^, 50.7 × 10^4^) *
iPAH n = 52	957.8 (738.1, 1117) *	20.7 (11.8, 29.7)	8.8 × 10^5^ (8.3 × 10^5^, 9.4 × 10^5^)	0.03 (0.02, 0.04) *	42 × 10^4^ (8.6 × 10^4^, 75.4 × 10^4^) *
PAH-SSc n = 31	1272 (847.9, 1695) *	28.9 (17.1, 40.8) *	7.8 × 10^5^ (6.4 × 10^5^, 9.3 × 10^5^)	0.04 (0.03, 0.05)	48.5 × 10^4^ (41 × 10^4^, 138 × 10^4^) *
PAH-HIV n = 20	661.4 (494.5, 828.3)	8.5 (5.0, 12.0)	8.7 × 10^5^ (7.3 × 10^5^, 10.2 × 10^5^)	0.03 (0.01, 0.04) *	5.8 × 10^4^ (1 × 10^4^, 12 × 10^4^) *
PAH-PoH n = 17	1179 (606.2, 1751) *	9.4 (6.1, 12.7)	9.0 × 10^5^ (7.3 × 10^5^, 10.8 × 10^5^)	0.02 (0.01, 0.03) *	5.9 × 10^4^ (2.4 × 10^4^, 9.3 × 10^4^) *
PAH-CTD n = 15	870.3 (586.6, 1154)	14.0 (6.9, 21.2)	7.4 × 10^5^ (5.7 × 10^5^, 9.0 × 10^5^)	0.03 (0.01, 0.04)	7.0 × 10^4^ (1.9 × 10^4^, 12.0 × 10^4^) *
PAH-h n = 9	698.6 (110, 1287)	14.2 (4.9, 23.5)	8.5 × 10^5^ (7.0 × 10^5^, 10.1 × 10^5^)	0.03 (0.01, 0.04)	24 × 10^4^ (−23.2 × 10^4^, 71.4 × 10^4^) *
SSc without PAH n = 44	2247 (1028, 3466) *	22.4 (8.3, 36.5)	7.0 × 10^5^ (5.0 × 10^5^, 9.1 × 10^5^)	0.01 (0.04, 0.05) *^#^	91 × 10^4^ (33.2 × 10^4^, 148 × 10^4^) *
HIV without PAH n = 22	1855 (1143, 2567) *^,^^†^	5.2 (2.8, 7.6) *	9.2 × 10^5^ (8.3 × 10^5^, 10.1 × 10^5^)	0.04 (0.04, 0.05)	6.9 × 10^4^ (3.2 × 10^4^, 10.6 × 10^4^) *^,#^

Values are expressed as mean, 95% confidence interval (CI). Definition of abbreviations: EMVs/µL PPP: endothelial microvesicles expressed per μL of poor platelet plasma (PPP); CL: healthy controls; PAH: pulmonary arterial hypertension; iPAH: idiopathic pulmonary arterial hypertension; PAH-SSc: PAH-associated with systemic sclerosis; HIV-PAH: PAH-associated with HIV; CTD-PAH PAH-associated with other connective tissue diseases; PAH-PoH: portopulmonary hypertension. * *p* < 0.05 compared to controls, ^†^ *p* < 0.05 compared to PAH-HIV, ^#^ *p* < 0.05 compared to PAH-SSc.

## Data Availability

All data relevant to the study are included in the article or uploaded as supplementary information.

## Supplementary Materials

The following are available online at https://www.mdpi.com/article/10.3390/cells10071688/s1, Figure S1: Gating strategy for endothelial microvesicles (EMVs) and for progenitor cells (PCs), Figure S2: Correlation between circulating endothelial microvesicles (EMVs) and progenitor cells (PCs), Figure S3: Number of circulating endothelial microvesicles (EMVs), percentage of circulating progenitor cells (PCs) and EMVs/PCs ratio in iPAH, PAH-PoH and PAH-HIV patients and healthy controls, Figure S4: Number of circulating endothelial microvesicles (EMVs) and percentage of circulating progenitor cells (PCs), Table S1: Clinical and hemodynamic characteristics in SSc and HIV patients with and without PAH and healthy controls, Table S2: Clinical characteristics and circulating EMVs and PCs count before and after PAH-targeted treatment, Table S3: Correlations between the number of PCs, EMVs and EMV/PC ratio and clinical features, Table S4: Differences in hemodynamic severity at basal and after 3 months PAH-targeted treatment considering increase or not increase of PCs.
