# Supplementary material for: Circulating Cell Biomarkers in Pulmonary Arterial Hypertension: Relationship with Clinical Heterogeneity and Therapeutic Response
Source: Cells. 2021 Jul 4;10(7):1688. doi: 10.3390/cells10071688 (PMC8304946; doi:10.3390/cells10071688)
Supplement: Supplementary file 1 [file cells-10-01688-s001.zip › cells-1225660-supplementary.pdf]

cells

MDPI

ONLINE RESOURCE

Circulating Cell Biomarkers in Pulmonary Arterial Hypertension: Relationship with Clinical Heterogeneity and Therapeutic Response

Running title: Biomarkers in pulmonary arterial hypertension.

Olga Tura-Ceide [1,2,3,*], Isabel Blanco [1,2], Jéssica Garcia-Lucio [1], Roberto del Pozo [1], Agustín Roberto García [1], Elisabet Ferrer [1], Isabel Crespo [4], Diego A. Rodríguez-Chiaradia [2,5], Carmen Pilar Simeon-Aznar [6], Manuel López-Meseguer [7], Clara Martín-Ontiyuelo [1], Víctor I. Peinado [1,2] and Joan Albert Barberà [1,2,*]

[1] Department of Pulmonary Medicine, Hospital Clínic-Institut d'Investigacions Biomèdiques August Pi i Sunyer (IDIBAPS), University of Barcelona, 08036 Barcelona, Spain; iblanco2@clinic.cat (I.B.); jss5@hotmail.com (J.G.-L.); rdelpozo81@gmail.com (R.d.P.); garcia83@clinic.cat (A.R.G.); elisabet.ferrer7@gmail.com (E.F.); clmartin@clinic.cat (C.M.-O.); vpeinado@clinic.cat (V.I.P.)
[2] Biomedical Research Networking Centre on Respiratory Diseases (CIBERES), 28029 Madrid, Spain; darodriguez@pardesalutmar.cat
[3] Girona Biomedical Research Institute (IDIBGI), 17190 Girona, Spain
[4] Flow Cytometry and Cell Sorting Facility, Institut d'Investigacions Biomèdiques August Pi i Sunyer (IDIBAPS), 08036 Barcelona, Spain; isabel.crespo@idibaps.org
[5] Pulmonology Department, Hospital del Mar, Institut Hospital del Mar d'Investigacions Mèdiques (IMIM), Universitat Pompeu Fabra (UPF), 08003 Barcelona, Spain
[6] Unitat de Malaties Autoimmunes Sistèmiques. Servei de Medicina Interna Hospital Universitari Vall d'Hebron, Departament de Medicina, Universitat Autònoma de Barcelona, 08035 Barcelona, Spain; cpsimeon@vhebron.net
[7] Servei de Pneumologia, Hospital Universitari Vall d'Hebron, Departament de Medicina, Universitat Autònoma de Barcelona, 08035 Barcelona, Spain; manuelopez@vhebron.net
* Correspondence: tura@clinic.cat (O.T.-C.); jbarbera@clinic.cat (J.A.B.); Tel.: 34-93-227-5747 (J.A.B.); Fax: 34-93-227-5455 (J.A.B.)

Keywords: Pulmonary arterial hypertension; endothelial dysfunction; biomarkers; progenitor cells; endothelial extracellular vesicles; PAH-specific treatment

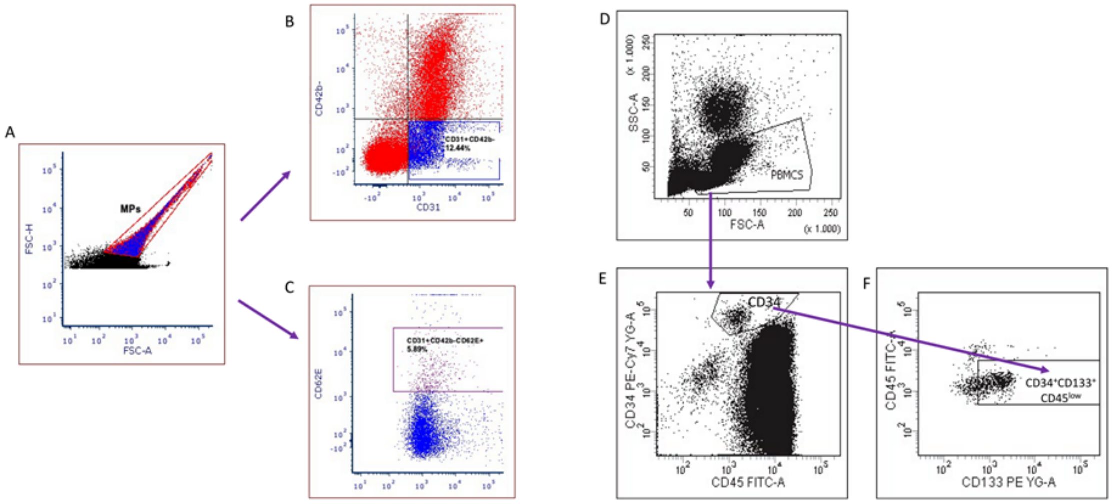

Figure S1. Gating strategy for endothelial microvesicles (EMVs) and for progenitor cells (PCs). A) MP analysis based on size and fluorescence; B) Sample analysed by two-colour fluorescence dot-plot as $CD31^{+}CD42b^{-}$ (total EMVs); C) sample analysed by three colour fluorescence dot plot as $CD31^{+}CD42b^{-}CD62E^{+}$ (activated EMVs). D) Peripheral blood mononuclear cells (PBMNCs) selection based on forward and side scatter; E) Sample analysed by two colour fluorescence dot plot as $CD34^{+}CD45^{low}$ cells and F) sample analysed by three-colour fluorescence dot plot as $CD34^{+}CD133^{+}CD45^{low}$ cells.

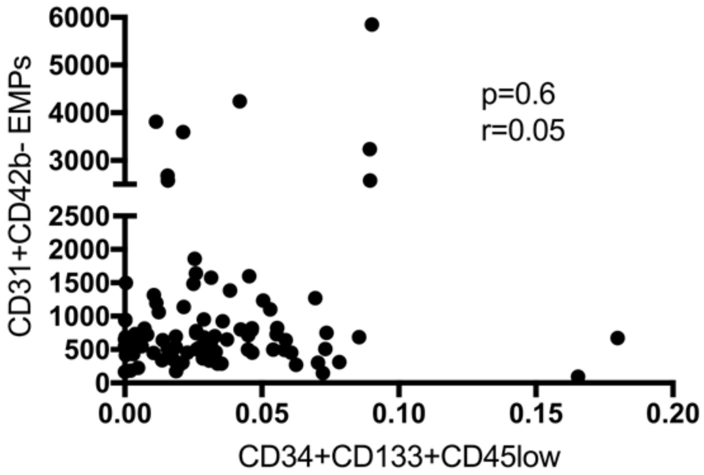

Figure S2 Correlation between circulating endothelial microvesicles (EMVs) and progenitor cells (PCs). Correlation between EMVs and PCs in all PAH patients. Spearman's rank correlation coefficient.

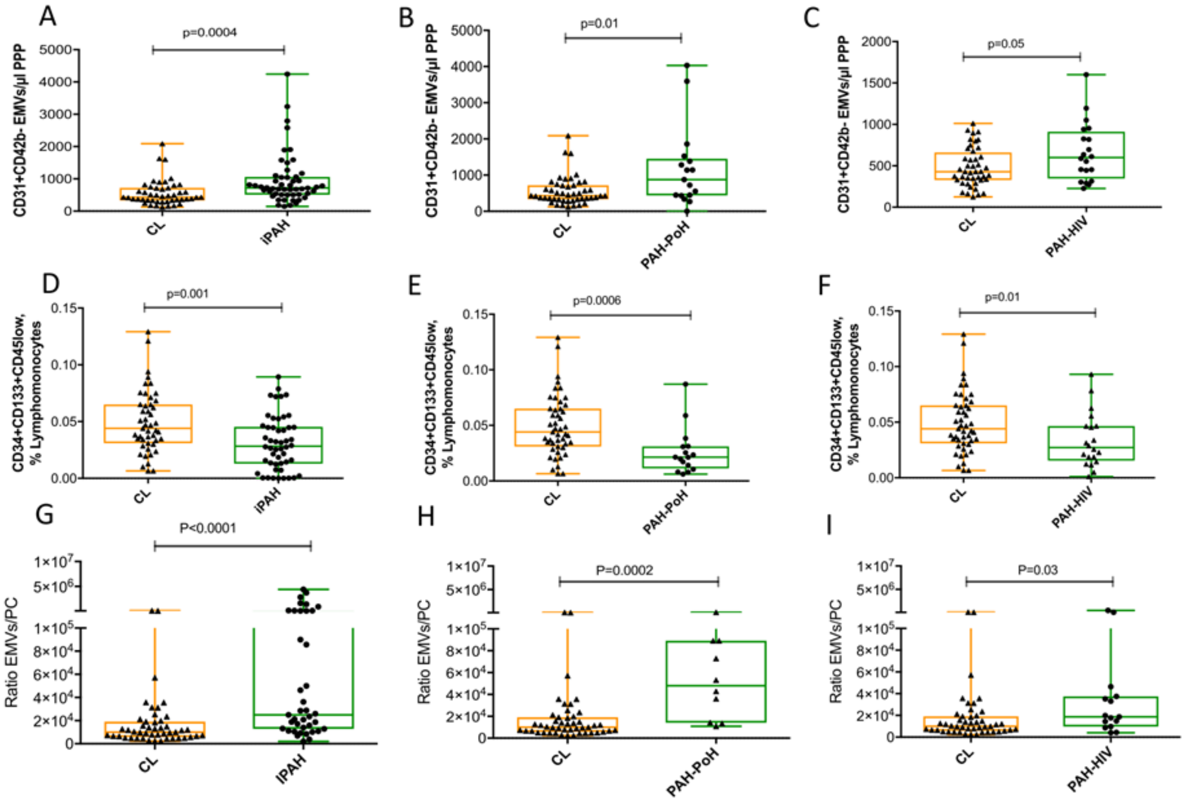

Figure S3. Number of circulating endothelial microvesicles (EMVs), percentage of circulating. progenitor cells (PCs) and EMVs/PCs ratio in iPAH, PAH-PoH and PAH-HIV patients and healthy controls. A) Number of EMVs in controls and iPAH; B) Number of EMVs in controls and PAH-PoH; C) B) Number of EMVs in controls and PAH-HIV; D) Percentage of circulating progenitor cells (PCs) in controls and iPAH; E) Percentage of circulating progenitor cells (PCs) in controls and PAH-PoH; F) Percentage of circulating progenitor cells (PCs) in controls and PAH-HIV; G) EMVs/PCs ratio in controls and iPAH; H) EMVs/PCs ratio in controls and PAH-PoH; I) EMVs/PCs ratio in. controls and PAH-HIV. The whiskers extend from the minimum to maximum points. Mann Whitney Rank Sum test, * $p < 0.05$.

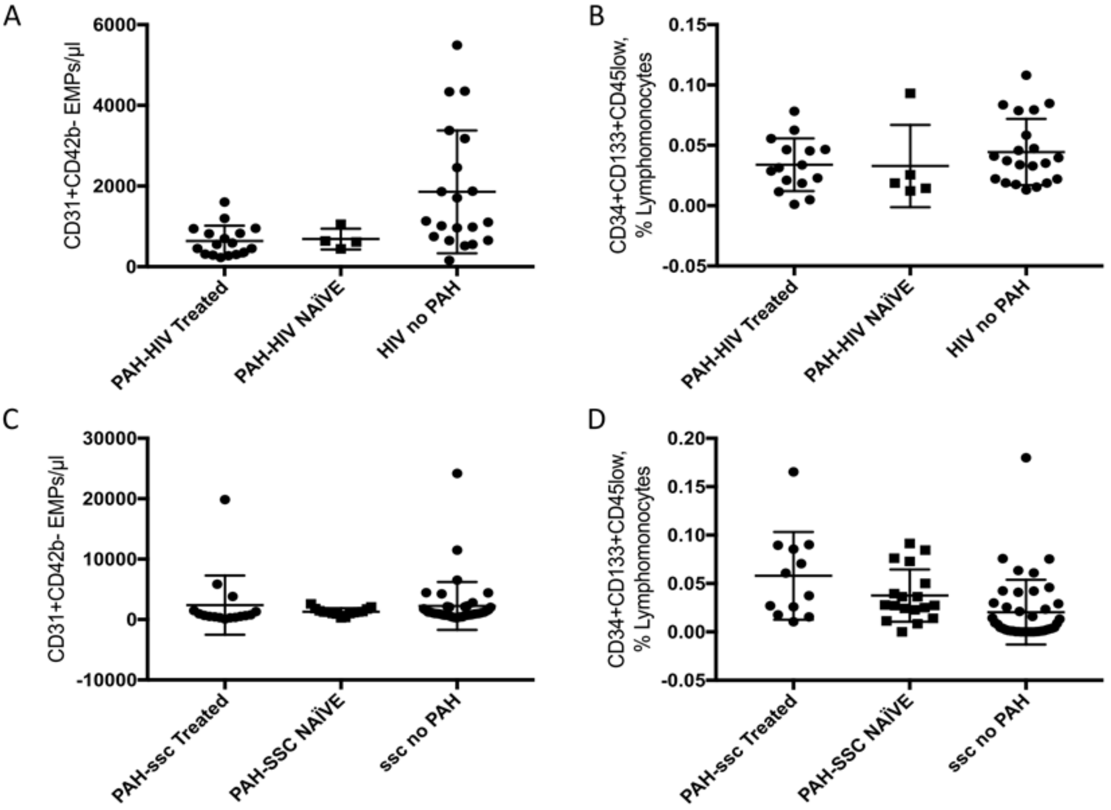

Figure S4. Number of circulating endothelial microvesicles (EMVs) and percentage of circula ing progenitor cells (PCs) in PAH-HIV (A-B) and PAH-SSc (C-D) with and without PAH. PAH-HIV (AB) and PAH-SSc patients (C-D) were separated into treatment naïve patients and prevalent patients receiving PAH-specific therapy. Kruskal-Wallis One Way Analysis of Variance, $p > 0.05$.

Table S1. Clinical and hemodynamic characteristics in SSc and HIV patients with and without PAH and healthy controls.

Variables	CL n=47	SSc n=44	SSc-PAH n=31	HIV n=22	HIV-PAH n=20
Age, years	48.0 ± 14.3	53.9 ± 10.1	64.4 ± 11.2†$	43.3 ± 9.3	46.2± 6.7
Male sex n (%)	20 (42.5%)	5 (11.3%)†	3 (9.6%)†	19 (86.3%)*	10 (50%)#
BMI (Kg/m^2)	25.3 ± 4.0	25.7 ± 4.4	26.3 ± 4.7	25.3 ± 4.0	23.0± 4.3
FVC(% predicted)	101.3 ± 12.6	100.4 ± 15.3	78.3 ± 18.9†$	105.8± 13.9	94.0± 16.2
FEV$_1$(% predicted)	102.8± 13.2	101.3 ± 12.8	77.3± 19.5†$	97.6± 16.2	83.5 ± 17.9*#
FEV$_1$/FVC (%)	83.6 ± 6.0	79.0 ± 5.3†	73.6 ± 6.4†$	74.5 ± 8.1*	70.0± 9.9*
TLC (% predicted)	103.2± 10.6	103.5± 16.0	84.2± 15.7†$	105± 13.3	98.7± 12.6
Dlco(% predicted)	91.9 ± 14.6	73.4 ± 12.2†	48.2 ± 16.8†$	87.8 ± 9.4	53.1± 9.9*#
DLCO/VA	101± 21.9	78.3 ± 12.5	63.9 ± 14.4†$	86.5± 14.4	58.6 ± 16.7*#
PaO$_2$ (mmHg)	ND	ND	63.9 ± 14.4	ND	76.5 ± 14.2
PAPm (mmHg)	ND	ND	42.4 ± 13.5*	ND	51.3 ± 13.7
PAWP (mmHg)	ND	ND	9.4± 3.1	ND	9.2± 4.3
RAP (mmHg)	ND	ND	9.3± 5.6	ND	7.4± 4.4
CI, (L.m^2.min^{-1})	ND	ND	2.3± 0.5	ND	2.3± 0.7
PVR (dyn.seg.cm^5)	ND	ND	682.3± 361.5	ND	894.5± 573.3
BNP (pg/ml)	16.8± 4.3	47.9± 51.5†	327± 314.2†$	18.5± 22.6	199.5± 296.5*#
WHO FC n, (%)					
I	47 (100)	43 (97.7)	1 (3.2)†$	18 (81.1)#	3 (15.0)*#
II	0 (0)	0 (0)	11 (35.4))	2 (9.0)	11 (55.0)
III-IV	0 (0)	1 (2.2)	19 (61.3)	2 (9.0)	6 (30.0)

Data are shown as mean± SD. Definition of abbreviations: PAH: pulmonary arterial hypertension; CL: healthy controls; BMI: body mass index; FVC: forced vital capacity; FEV$_1$: forced expiratory volume in the first second; TLC: total lung capacity; DLco: diffusing capacity of the lung for carbon monoxide; PaO$_2$: arterial partial oxygen pressure; mPAP: mean pulmonary arterial pressure; PAWP: pulmonary artery wedge pressure; RAP: right atrial pressure; CI: cardiac index; PVR: pulmonary vascular resistance; BNP: brain natriuretic peptide, WHO FC: functional class world health organization; and ND: not determined. *$p<0.05$ HIV-PAH compared to controls, #$p<0.05$ HIV-PAH compared to HIV, †$P<0.05$ SSc with or without PAH compared to CL, $ $P<0.05$ SSc-PAH compared to SSc, Kruskal-Wallis test followed by post-hoc Dunn´s multiple comparisons test.

Table S2. Clinical characteristics and circulating EMVs and PCs count before and after PAH-targeted treatment.

	Pre-treatment	Post-treatment	P-value	n
**6MWD, mts**	343 ± 148	392 ± 147	0.005*	53
**BNP, pg/mL**	311 ± 327	144 ± 171	<0.001*	53
**NYHA FC III-IV, n (%)**	32 (59.3)	18 (33.3)	0.02 *	53
**mPAP, mmHg**	51 ± 16	45 ± 13	0.42	12
**CI, L/min/m^2**	2.3 ± 0.6	2.8 ± 0.5	0.04 *	12
**RAP, mmHg**	8 ± 4	5 ± 2	0.16	12
**PVR, dyn.s.cm^{-5}**	863 ± 412	518 ± 289	0.04 *	12
**PCs**	0.03 ± 0.02	0.03 ± 0.02	0.97	53
**EMVs**	1050 ± 697.5	1359 ± 1061	0.15	53
**Ratio EMVs/PCs**	3.9x10^4 ± 3,4x10^4	5.5x10^4 ± 6.0x10^4	0.45	53

Data are shown as mean± SD. Definition of abbreviations: 6MWD: six-minute walking distance; BNP: brain natriuretic peptide; NYHA FC: New York heart association functional class; mPAP: mean pulmonary arterial pressure; CI: cardiac index; RAP: right atrial pressure; PVR: pulmonary vascular resistance; PCs: progenitor cells; EMVs: endothelial microvesicles. Student's t-test for normally distributed variables, or Mann Whitney Rank Sum test for non-normally distributed variables, *$p<0.05$.

Table S3. Correlations between the number of PCs, EMVs and EMV/PC ratio and clinical features

	mPAP (mmHg)	CI (L/min/m^2)	RAP (mmHg)	PVR (dyn.seg.cm^{-5})	6MWD (mts)	BNP (pg/ml)	DLCO (%)	NYHA-FC
**PCs**	r=0.17, p=0.2	r=0.25, p=0.1	r=0.02, p=0.86	r=025, p=0.1	r=0.09, p=0.55	r=0.21, p=0.18	r=-0.22, p=0.15	r=0.14 p=0.33
**EMVs**	r=0.08, p=0.6	r=0.05, p=0.7	r=0.08, p=0.58	r=0.01, p=0.94	r=-0.07, p=0.65	r=0.17, p=0.28	r=-0.14, p=0.36	r=0.12 p=0.41
**EMVs/PC ratio**	r=-0.10, p=0.52	r=0.29, p=0.06	r=0.08, p=0.59	r=-0.23, p=0.13	r=0.05, p=0.76	r=-0.05, p=0.76	r=0.16, p=0.30	r=-0.09 p=0.54

Definition of abbreviations: PCs: progenitor cells; EMVs: endothelial microvesicles. mPAP: mean pulmonary arterial pressure; RAP: right atrial pressure; CI: cardiac index; PVR: pulmonary vascular resistance; 6MWD: six-minute walking distance; BNP: brain natriuretic peptide; DLco: diffusing capacity of the lung for carbon monoxide; NYHA-FC: New York heart association functional class. Spearman's rank correlation coefficient.

Table S4: Differences in hemodynamic severity at basal and after 3 months PAH-targeted treatment considering increase or not increase of PCs.

	Increase PCs (n=5)			No increase PCs (n=7)		
	**Basal**	**Follow-up**	**p-value**	**Basal**	**Follow-up**	**p-value**
**mPAP, mmHg**	49 ± 7	45 ± 14	0.522	52 ± 20	43 ± 13	0.434
**PVR, UW**	11,6 ± 3.7	5.5 ± 1.6	0.083	10.1 ± 7.2	7.3 ± 4.7	0.349
**CI, L/min/m^2**	2.06 ± 0.43	3.05 ± 0.4	0.004 *	2.57 ± 0.6	2.80 ± 0.76	0.166
**RAP, mmHg**	6 ± 3	10 ± 6	0.401	6 ± 3	7 ± 3	0.252

Definition of abbreviations: PCs: progenitor cells; mPAP: mean pulmonary arterial pressure; PVR: pulmonary vascular resistance; CI: cardiac index; RAP: right atrial pressure. Student's t-test *$p<0.05$.
